# Antimicrobial Peptides: Powerful Biorecognition Elements to Detect Bacteria in Biosensing Technologies

**DOI:** 10.3390/molecules23071683

**Published:** 2018-07-10

**Authors:** Mireia Hoyos-Nogués, F. J. Gil, Carlos Mas-Moruno

**Affiliations:** 1 Biomaterials, Biomechanics and Tissue Engineering Group (BBT), Department of Materials Science and Metallurgical Engineering, Universitat Politècnica de Catalunya (UPC), 08019 Barcelona, Spain; mireia.hoyos@upc.edu (M.H.-N.); xavier.gil@uic.cat (F.J.G.); 2 Barcelona Research Center in Multiscale Science and Engineering, UPC, 08019 Barcelona, Spain; 3 Universitat Internacional de Catalunya (UIC), 08195 Sant Cugat del Vallès, Spain

**Keywords:** antimicrobial peptides, bacteria, biofilm, bacterial detection, biosensors, impedance

## Abstract

Bacterial infections represent a serious threat in modern medicine. In particular, biofilm treatment in clinical settings is challenging, as biofilms are very resistant to conventional antibiotic therapy and may spread infecting other tissues. To address this problem, biosensing technologies are emerging as a powerful solution to detect and identify bacterial pathogens at the very early stages of the infection, thus allowing rapid and effective treatments before biofilms are formed. Biosensors typically consist of two main parts, a biorecognition moiety that interacts with the target (i.e., bacteria) and a platform that transduces such interaction into a measurable signal. This review will focus on the development of impedimetric biosensors using antimicrobial peptides (AMPs) as biorecognition elements. AMPs belong to the innate immune system of living organisms and are very effective in interacting with bacterial membranes. They offer unique advantages compared to other classical bioreceptor molecules such as enzymes or antibodies. Moreover, impedance-based sensors allow the development of label-free, rapid, sensitive, specific and cost-effective sensing platforms. In summary, AMPs and impedimetric transducers combine excellent properties to produce robust biosensors for the early detection of bacterial infections.

## 1. The Burden and Risks of Bacterial Infections

Bacteria mainly survive as multicellular aggregates known as biofilms, which are involved in at least two thirds of all infections and exhibit rising adaptive resistance to conventional antibiotic therapies [1]. Biofilm infections require complex and costly clinical treatments and can lead to morbidity and mortality in patients. Specifically, the World Health Organization (WHO) identified lower respiratory tract infections as the fourth-highest cause of death worldwide, with diarrheal diseases (ninth), and tuberculosis (tenth) being among the top 10 leading causes of death in 2016 [2]. 

Among patients that are vulnerable to suffer an infection, those bearing medical devices such as catheters, artificial heart valves, prosthetic joints, and other implants, are more susceptible to develop a biomaterial associated infection (BAI). One factor contributing to the onset of BAI is the decreased efficacy of the local immune defense induced by a foreign body (i.e., the medical device). In agreement, the number of bacteria required to cause an infection is significantly lower in the presence of an implant. Another important factor is the predilection of bacteria to adhere to a substrate, where they can replicate, and form a biofilm from which they can invade the surrounding tissues and cause an infection. Conventional antibiotic therapy often fails due to the low levels of antibiotic at the site of infection, and, consequently, removal of the biomaterial is the last option to control the infection [3]. This underlines the pressing need for more specific and faster alternative strategies that can be employed to diagnose and prevent BAI [4]. 

Therefore, the early detection and identification of bacterial pathogens remain a high priority goal. Biofilms form and spread rapidly, thus a fast detection often means the difference between life and death for the patient. Moreover, specificity is also needed to select an appropriate clinical treatment or to carefully assess the danger to the public [5].

The identification and quantification of bacteria has traditionally been performed using a variety of time-consuming methods, which required specialized laboratories and costly equipment. Typically, analytes (e.g., blood, saliva, urine, or food samples) are analyzed using various techniques, namely, microscopical observation, cell culture, biochemical and immunological tests, or genetic analysis. However, all methods have limitations. Microscopy is relatively quick but not specific, and requires the staining of bacteria. Culturing and growing bacteria on agar can take up to several days. Furthermore, not all bacteria can be cultured in the laboratory [4]. Biochemical assays and immunological tests (such as ELISA) are good methods to detect specific bacterial markers. However, these methods are time consuming and costly, and require skilled personnel to run the tests and interpret the results [5].

Further testing by means of genetic analysis has enabled a more efficient identification of bacterial strains. PCR is a highly sensitive technique that relies on the use of bacterial genetic material. It does not require culturing bacteria due to the small sample needed, but it is still a tedious and expensive procedure, which can last for days. Real-time PCR analysis can be completed faster, within several hours, but it still depends on specialized equipment (costly) and trained users. Moreover, it entails sample enrichment and purification prior to analysis (complex) [6].

Hence, there is an urgent demand for more rapid, cost-effective, and sensitive methods, which can detect and identify bacteria or its components. In this regard, biosensors have recently been looked upon as attractive alternatives to the existing methods to detect pathogens. Biosensors show excellent properties, including high sensitivity, specificity and reproducibility, without the need for sample preparation steps. Furthermore, they are cheap and fast, which would allow the doctor to quickly ascertain an infection and prescribe an appropriate treatment [4,5,6,7,8].

The versatility of biosensors is also illustrated by the large number of targets they can detect, including bacteria [5,9], viruses [10] and a variety of biomolecules such as specific DNA sequences [11], proteins [12,13] and peptides [14].

The aim of this review is to present the state of the art and recent advances in the field of biosensors for bacterial detection. We will particularly focus on the use of antimicrobial peptides (AMPs) as biorecognition moieties for the electrochemical (impedance-based) detection of bacteria. 

## 2. Biosensors for Detecting Bacterial Cells

A biosensor is defined as any measuring device that incorporates a biological entity in order to recognize a target molecule and produce a detectable signal. Biosensors combine the outstanding selectivity of biological interactions found in nature with the processing power of modern microelectronics, and are nowadays used in a variety of fields including medicine, environmental studies, food and processing industries. 

**Figure 1:**
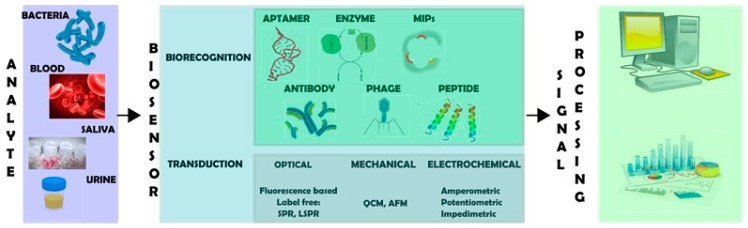
Schematic representation of the biosensing process. A biosensor has two main components: a biorecognition moiety that interacts with analytes and a transducer that converts such interaction into a measurable signal.

A biosensor typically encompasses two major components, a biorecognition moiety and a transduction platform (Figure 1), together with an amplifier system and a signal processer [8,15]. Biorecognition elements are commonly biomolecules such as antibodies, enzymes or peptides that are able to recognize and interact with high specificity with a target analyte. Upon interaction, a catalytic or binding event is produced. This process is converted by the transduction platform into a measurable signal, which is proportional to the analyte concentration.

Response time, dynamic range, limit of detection (LoD), single-to-noise ratio, and specificity are important features that need to be optimized to obtain an adequate biosensor. These parameters are strongly related to the elected biological sensing element, the transducer and the signal amplifier [9]. Thus, to efficiently detect bacteria, it will be crucial to select biorecognition elements with high affinity for bacterial components, and an appropriate system of transduction to ensure detectable signals are generated, even at very low concentrations of bacteria. The following sections will cover both elements. 

Additionally, it should be noted that there are other aspects that can influence the sensitivity and specificity of a biosensor. In particular, material-dependent unspecific bacterial adhesion as well as adhesins and bacteria-secreted extracellular polymeric substances (EPS) may greatly limit the detection efficiency and performance of the sensor. For example, it has been observed that microorganisms attach with higher preference to hydrophobic surfaces and plastics such as Teflon, compared to more hydrophilic substrates like glass or metal [16]. Moreover, it has also been described that most bacteria produce EPS and adhesins to promote bacterial attachment [17,18]. This means that bacteria may attach to the biosensor surface regardless of the biorecognition element. Such non-specific attachment is often neglected in biosensor studies and emphasizes the importance of utilizing low fouling surfaces (e.g., polymers like polyethylene glycol) to block unspecific protein and bacterial adhesion [19]. 

Two classes of biosensors have been developed to detect bacteria: (i) those which require sample processing (e.g., bacterial lysis) to liberate bacterial components, and (ii) processing-free systems, which target whole bacteria. In the first category, bacterial components such as DNA [11], RNA [20], coagulation factors [12], and exotoxins [21] can be detected. Its main disadvantage is the requirement for sample processing, which increases the time and cost of the analysis. Therefore, biosensors allowing the direct detection of whole bacteria without the need of extra pre-treatments or reagents are a preferred strategy, as they are generally faster and more cost-effective. In this review we will focus on label-free sensors capable of detecting whole bacteria.

### 2.1. Biorecognition Elements 

As previously explained, biorecognition moieties are crucial elements in a biosensor (Figure 1), as they serve to identify the target analyte and determine the efficiency of the biosensor in terms of both sensitivity and specificity. Thus, the choice of the recognition element will strongly influence the performance of the sensor. Besides a high binding affinity for the target analyte, these elements should also have a good stability [22]. 

**Figure 2:**
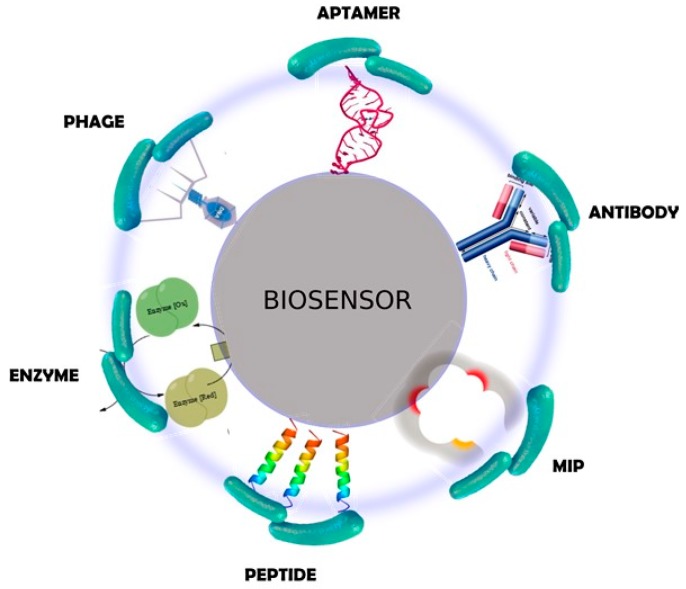
Schematic representation of different types of biorecognition elements for bacterial detection.

The list of biological entities that can be used to develop a biosensor is expanding rapidly, as the technology of biosensing methods advances. For bacterial detection, virtually any molecule that recognizes or attaches to a bacterial cell can potentially be used in a biosensor. The variety of elements ranges from small molecules, such as sugars and short peptides, to large molecules including proteins, and even viruses and cells. Representative examples are schematically shown in Figure 2. A comprehensive classification can be found in the recent literature [22,23].

Biorecognition probes can be classified according to different criteria. If the origin is selected as such criterion, two major categories can be used: those elements found in nature, and those designed and engineered by men. Obviously, overlapping between these two families exist, and many strategies often focus on recognition elements from nature that are modified in the lab. There is no perfect candidate. Biological elements such as cells and large proteins typically show higher specificity for bacteria than small biomolecules, but their complexity is accompanied by reduced stability and difficulties in handling. Synthetic molecules, on the contrary, are useful in a way that their binding affinity can be optimized, while retaining good stability under testing conditions. 

Enzymes and antibodies are two common examples of biorecognition elements from natural origin and are widely used in biosensing technologies. While enzymatic biosensors rely on the reaction of an enzyme with its substrate (e.g., the substrate is metabolized by the enzyme or it inhibits the enzymatic reaction), immunosensors (sensors coated with antibodies) interact with antigens present on microbial surfaces. Both approaches have good levels of specificity and can be produced at relatively low cost. However, they have limitations in terms of storage, handling and stability. Specific problems also exist for each system. Enzymatic biosensors commonly contain only one enzyme and cannot be used for the detection of whole bacteria, and achieving a good signal enhancement for small molecules is not always possible with antibodies [24]. 

Lectin/carbohydrate recognition can also be exploited to detect bacteria. Lectins, which are proteins that recognize carbohydrates, can be used to specifically react with bacterial carbohydrates [25]. It should be noted that the opposite approach, i.e., functionalizing the sensor with sugars and detecting bacterial lectins, is also possible [23] In this regard, carbohydrate-based sensors are an attractive alternative due to the chemical stability of carbohydrates and good grafting properties. Nonetheless, the detection of bacterial species using these molecules can produce false positives in complex samples because several lectins can bind different carbohydrates, as well as different carbohydrates can bind the same lectin, thus causing a significant reduction in specificity.

More complex entities, e.g., living systems, represent alternative biorecognition elements. This is the case of human cells. Mammalian cells express a wide range of proteins and receptors to perceive their environment, which make them appealing bioreceptors to sense bacteria [21,26,27]. Bacteriophages may fall within this category as well. Bacteriophages, or simply phages, are viruses that infect and replicate within bacteria. As such, they possess highly specific mechanisms to recognize bacteria and can be used for bacterial detection [23,28]. Interestingly, a key feature of phage-based sensors is their ability to distinguish between viable and inactive bacteria, as they can only replicate within viable bacterial cells. Despite the advantages offered by these two approaches, several limitations are associated to their use, including their particularly difficult handling and immobilization on the biosensor, together with their low stability over time [29,30].

The last example of this category is antimicrobial peptides (AMPs). AMPs are members of the innate immune system of many organisms and, hence, are very effective in killing a wide range of pathogens, including Gram positive and Gram negative bacteria, viruses, fungi and even cancerous cells [31]. Relatively short in size and generally cationic and amphipathic, these peptides commonly interact with bacterial cell membranes through electrostatic interactions, and subsequently disrupt membrane integrity via hydrophobic contacts [32]. This bactericidal mechanism is less likely to be overcome by bacterial resistance than conventional antibiotic treatment, and for this reason AMPs are being considered as very promising therapeutic targets [33]. In addition to their broad biological potential, AMPs show intrinsic stability and can be produced by synthetic methods in large quantity [34]. It is thus not surprising that these molecules have also been used as bioreceptors for the sensing of bacteria [35,36], as they show clear advantages over the limitations of other methods. In this review, we will focus on AMPs as biorecognition elements. Their potential and features will be covered with greater detail in Section 3.

As previously commented, a second class of biorecognition elements would be those that have been engineered to very effectively recognize bacteria but with higher stability than classical recognition elements such as enzymes or antibodies. These strategies include the use of molecularly-imprinted polymers (MIPs) [37,38]; phage peptides, which recapitulate only the receptor binding protein (RBP) localized on the phage tail and responsible for bacterial recognition [30]; affibodies or engineered antibody mimetics with high target affinity [36,39]; and aptamers, short oligonucleotide molecules that unlike their natural counterparts (i.e., DNA and RNA) can bind to whole bacteria. Aptamers are produced by repeated rounds of in vitro selection using systematic evolution of ligands by exponential enrichment (SELEX) [40]. This method allows engineering aptamers with high affinity and specificity for bacteria [41]. These and other approaches have potential to replace classical strategies to develop novel biosensors with improved performance. However, their use as biosensing motives is still in some cases at an early stage of research and further studies in this direction are required.

### 2.2. Transduction Systems

**Figure 3:**
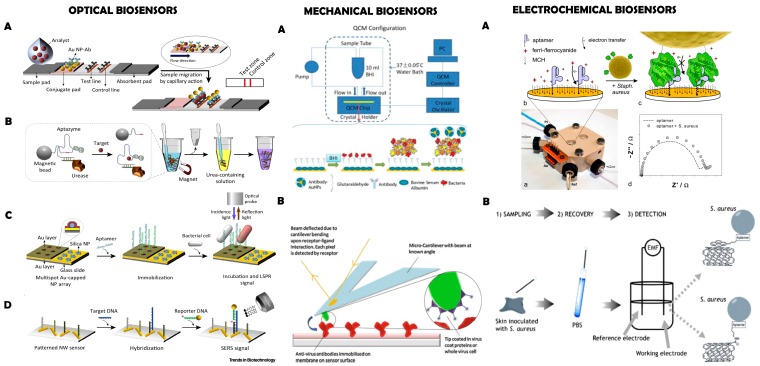
Three types of biosensors. OPTICAL BIOSENSORS: (**A**) lateral flow assay (LFA)-based biosensor; (**B**) solution-based colorimetric biosensor; (**C**) localized surface plasmon resonance (LSPR)-based biosensor; (**D**) surface-enhanced Raman scattering (SERS)-based biosensor. (NP: nanoparticle). Reprinted from [42], with permission from Elsevier. © 2015 Elsevier Ltd. MECHANICAL BIOSENSORS: (**A**) Nanoparticle-functionalized piezoelectric biosensor – quartz crystal microbalance (QCM) system. Reprinted from [50], with permission from Elsevier. © 2012 Elsevier B.V.; (**B**) Antibody conjugated microcantilever using atomic force microscopy (AFM) tapping mode. Reprinted from [6], with permission from Elsevier. © 2010 Elsevier B.V. ELECTROCHEMICAL BIOSENSORS: (**A**) Aptamer functionalized impedimetric biosensor. Figure reproduced from [71]; (**B**) Potentiometric biosensor. Reprinted from [70], with permission from Elsevier. © 2011 Elsevier B.V.

Once bacteria have been recognized by the biosensing moiety, it is necessary to convert this interaction into a measurable signal. This is achieved by a suitable transduction platform (Figure 1). There are many different types of transduction systems, which can be categorized according to the nature of the signal transduced. Electrochemical, optical, mechanical and thermal transducers are the most common types (Figure 3).

Impedimetric and optical sensors are nowadays frequently used to detect whole bacteria [4,23,42]; however, multiple combinations between bioreceptors and transducers are possible, giving rise to a very large number of biosensors for whole bacteria detection (Table 1). The following section briefly describes the main families of transducers used in the field, together with relevant examples of biosensing.

#### 2.2.1. Mechanical Transducers

Mechanical biosensors commonly operate measuring variations in the vibrational frequency of a piezoelectric crystal, which can be correlated to small changes in mass, e.g., the binding of a bacteria to a surface [106]. Mechanical biosensors are label-free and have high sensitivity and fast processing times. Among the large quantity of mechanical sensors existing at present [107], pathogenic bacteria are mainly detected using quartz crystal microbalance (QCM) or cantilever technology (Figure 3). 

QCM sensors have been combined with various types of bioreceptors to detect whole bacterial cells (Table 1), including antibodies [48,49,50,51], DNA molecules [67], lectins [73,74], AMPs [95,96] or phages [82]. The potential of AMPs has been highlighted in recent examples. For example, Shi et al. reported a biosensor using the AMP pleurocidin combined with single-walled carbon nanotubes (SWCNT) on a multichannel quartz crystal [96]. This AMP provided a broad spectrum of antibacterial activity with almost no effect on eukaryotic cells (e.g., erythrocytes). Interestingly, the interaction of pleurocidin with bacteria was associated with the detachment of the AMP from the SWCNT, resulting in a measurable change in the sensor frequency. This method allowed a real-time, sensitive and fast (only 15 min) detection of bacteria.

The second type of mechanical transducers is based on microcantilever technology. This technique is label-free, very sensitive, fast and can be miniaturized. Therefore, these transducers are ideal candidates for the development of point-of-care biosensors. Bacterial detection with microcantilevers has been described using either antibodies [52] or DNA [68] as biorecognition elements. Nevertheless, the performance of these sensors in complex matrixes has not been reported yet [4]. Noteworthy, the recently developed piezoelectric-excited millimeter-size cantilevers (PEMC) coupled to antibodies have been able to detect *Listeria monocytogenes* at infectious doses, both in PBS buffer and milk samples [53].

In addition to the well-known QCM and cantilever methods, nanowire-based sensors are emerging as new platforms with very high sensing potential (i.e., single molecule detection) [108]. For instance, nanowire arrays mechanical sensors were recently described to study bacterial adhesion at the single cell level [18]. This technique does not rely on the use of biorecognition elements, although the nanowires can be functionalized with proteins like adhesin to enhance bacterial attachment forces. Such new sensing platforms are interesting because they open the way to dissect the mechanisms of bacterial attachment at the biophysical level.

#### 2.2.2. Optical Transducers

Optical biosensors detect changes in the properties of light as a result of the interaction between an analyte and a bioreceptor. They allow highly specific and sensitive sensing of bacteria in a rapid, real-time and cost-effective fashion. In general, they can be divided into label-based (e.g., fluorescent) and label-free methods [109,110]. Plasmonic biosensors, relying on the use of surface plasmon resonance (SPR) or surface enhanced Raman spectroscopy (SERS), are nowadays frequently used for pathogen detection **(Figure 3**) [42]. These transducing systems can be combined with diverse bioreceptors including antibodies, phages and lectins to efficiently detect whole bacteria (Table 1). For example, SPR-based sensors containing different lectins as recognition units were able to detect *E. coli* 0157:H7 with a LoD of 3 × 10^2^ CFU/mL [72]. Interestingly, the detection of multi-resistant pathogens has been achieved using phages, sensing bacteria at concentration of 10^3^ CFU/mL in less than 20 min [78,79], and with antibodies specific to antimicrobial resistance-related protein (e.g., anti-penicillin binding protein 2A, PBP2a) [47]. SERS has also been widely used to detect pathogens due to its single molecule-level sensitivity and molecular specificity [111,112]. The main limitation of this method is the need of specialized software and a database with SERS spectra of bacteria, which has prevented its widespread use.

Another technique derived from SPR is localized SPR (LSPR), which involves a resonant oscillation at the surface of a metal nanoparticle under the incidence of an external light. Due to the unique properties of LSPR, gold nanoparticles (AuNPs) modified with antibodies have been widely used to develop bacterial biosensors [46,65]. However, commercialization of this technique has been limited because it requires skilled operators and sophisticated instruments. 

Colorimetric assays are an attractive alternative to overcome the aforementioned limitations and can be developed into portable, easy-to-use, and user-friendly devices for in situ analysis. Examples of colorimetric sensing of bacteria at low concentrations include the use of cationic AuNPs bound to enzymes [43] or paper-based analytical devices (μPAD) [44]. A variety of DNA aptamers coupled to quantum dots or to magnetic nanoparticles (MNPs) were also developed against different bacterial strains with high sensitivity [63,64]. Other described combinations are phage-based magnetoelastic (MES) biosensors [80,81] and aptazymes combined with magnetic beads [66]. The use of AMPs as biorecognition elements in optical devices is usually based on fluorescence microscopy [91,92,93,94]. For instance, magainin I-coated glass microspheres (GMs) on a microfluidic sensor were used to detect *E. coli* with very good efficiency [93]. This was achieved owing to the high specific surface area provided by GMs, which ensured a great number of AMP-bacteria interactions.

#### 2.2.3. Electrochemical Transducers

Electrochemical biosensors represent the most widespread class of sensors for bacterial biosensing, and are based on the use of current, potential and impedance changes to transduce the biological recognition event. Compared to other analytical techniques, electrochemical detection has the advantage of being inexpensive, robust, fast and relatively simple to operate. Another interesting feature is that in general this method does not require sample preparation as the biological sensing is highly selective and the signal is provoked by electrochemical and physical changes on a conducting polymer layer. Electrochemical biosensors usually contain three electrodes (a reference electrode, a working electrode, and a counter electrode) and are classified according to the electrical parameter measured as (i) amperometric, (ii) potentiometric or (iii) impedimetric types.

Amperometric biosensors operate by generating a current signal when a potential is applied between the working and the reference electrodes. The current depends on the analyte concentration. Enzyme-based amperometric sensors were reported 40 years ago for the detection of glucose [113], but now can be applied to sense a wide range of analytes, including whole bacteria [54]. As shown in Table 1, in addition to enzymes, amperometric sensors can be developed by combination with other bioreceptors such as antibody-conjugated magnetic particles [54,55,62] or phages [87], among others. 

Potentiometric biosensors are based on the potential difference (voltage) between the working and reference electrodes. The electrical potential is proportional to the analyte concentration, which is compared to a reference potential (reference electrode). Thus, this technique relies on the sensitivity and selectivity of the working electrode and requires a stable and accurate reference electrode. Although potentiometry is widely used in the biosensor field, examples of potentiometric biosensors for the detection of whole bacterial cells are scarce. This is due to the fact that, unlike other methods such as impedance, potentiometry cannot provide specific and sensitive signals for large analytes such as bacteria. However, some innovative approaches using aptamers as bioreceptors have resulted in reasonably good LoD [69,70].

Finally, impedimetric biosensors measure changes in impedance over a suitable frequency range. In this case, the analyte interaction is translated into a change in the resistance and/or double-layer capacitance [114]. Compared to amperometric and potentiometric methods, sensors based on impedance have important advantages for the detection of whole bacteria: they are label-free, economic, highly sensitive, and can be miniaturized. Moreover, they are not affected by the presence of other compounds in the sample matrix [6,9]. Their main disadvantage is that any charged molecule can lead to a change in conductivity, thus affecting the selectivity of the sensor [15]. The typical experimental setup used in impedance biosensors is a three-electrode configuration, where the test signal is applied between the working and reference electrodes, while the current is measured at the counter electrode [115]. A large number of impedimetric biosensors for whole bacteria detection have been described in recent years (Table 1). The most common bioreceptors employed in this case are enzymes coated on nanoparticles [45], antibodies [14,57,58,59,60], aptamers [71], lectins [75,76,77] and phages [88,89]. The use of AMPs represents the main focus of this review and will be covered comprehensively in the following sections.

### 2.3. Signal Amplifiers

Signal amplification is used to improve the sensitivity of biosensors towards the detection of the desired target, e.g., bacterial cells. In this sense, the use of nanomaterials such as nanoparticles has drawn interest due to their remarkable optical and electrical properties (good conductivity and photoelectrochemical activity) and large surface/volume ratio. Nanomaterials can be used to recreate an optimal microenvironment that retains the biologically-active conformation of the molecules or to present multiple copies of the bioreceptor, assuring an optimal signal transduction and the stability of the biosensor [29]. This strategy can be exploited to obtain sensors with high affinity to detect a large number of analytes, including bacterial components and whole bacteria.

Examples of (nano-)materials that can be used as signal amplifiers for bacterial detection are carbon nanotubes (CNT), bio-compatible metal/metal oxide nanoparticles (e.g., AuNPs), gold nanorods, quantum dots, graphene-based materials and magnetic nanoparticles [22,98,116]. For example, a common strategy of signal amplification is to immobilize enzymes or antibodies on nanoparticles/nanobeads (Table 1) [49,61]. It is expected that the unique properties offered by these nanomaterials will allow developing of a new generation of nanostructured devices with increased potential to detect bacteria [23,24].

## 3. AMP-Based Impedimetric Biosensors

The next and last section of this review will focus on biosensors that contain AMPs as biorecognition molecules and rely on impedimetric signal transduction. Such types of biosensors have unique properties and are gaining increased popularity in the field. However, other combinations are also frequent and possible—examples have already been cited in the previous sections and in Table 1.

### 3.1. AMPs as Biorecognition Elements

As previously introduced, AMPs, also known as host defense peptides, are peptides produced by a large number of living systems to fight against invading pathogens. Their clinical use to replace antibiotics is attracting interest, as they have a broad spectrum of antibacterial activity but do not promote bacterial resistance. Moreover, they have shown potential to modulate the immune response and promote wound healing [33]. From a structural point of view, they are short (generally < 40 amino acids), cationic, amphipathic (they combine positively charged residues with hydrophobic ones) and can adopt diverse secondary structures [31,117]. 

Although AMPs have diverse mechanisms of antibacterial action, their major targets are cytoplasmic membranes. In general, they establish electrostatic interactions with lipopolysaccharides (LPS) and other negatively charged molecules on bacterial membranes, after which hydrophobic contacts with the lipidic components result in pore formation, disruption of the membrane and cell lysis [32]. The efficiency of AMPs in recognizing the phosphate groups of LPS has been exploited to develop biosensing assays to detect, classify, and quantify bacteria [36]. In some cases, AMPs are also internalized and inhibit vital processes such as protein transcription by binding to intracellular molecules [32].

From an engineering perspective, AMPs have also desirable properties to design biosensors. They can be synthesized with current solid-phase peptide synthesis methods in an automated fashion at low cost and in large quantities. Furthermore, they can be derivatized with chemical groups (i.e., spacers and anchors) to selectively functionalize diverse surfaces. The incorporation of chemoselective anchoring groups, together with the small molecular size of the peptides, allows an efficient immobilization on the sensor surface with high ligand density. Last but not least, AMPs are more stable under harsh environments than other biomolecules commonly used such as enzymes or antibodies [34].

However, it should be noted that the use of AMPs is not exempt from limitations. Bacterial detection in real, complex samples with AMPs is not always possible. Moreover, achieving sensitivity and, especially, selectivity within different bacterial strains is challenging and requires further investigations.

### 3.2. EIS Technique

Electrochemical impedance spectroscopy (EIS) is the most common technique among impedimetric methods. This technique measures the impedance of the system, by means of a scan over a wide range of potential frequencies. Changes in impedance values respond to physicochemical changes derived from the binding between the analyte and the bioreceptor. EIS measurements can be performed with a different number of electrodes in different configurations, usually named as two-, three- and four-electrode implementations.

**Figure 4:**
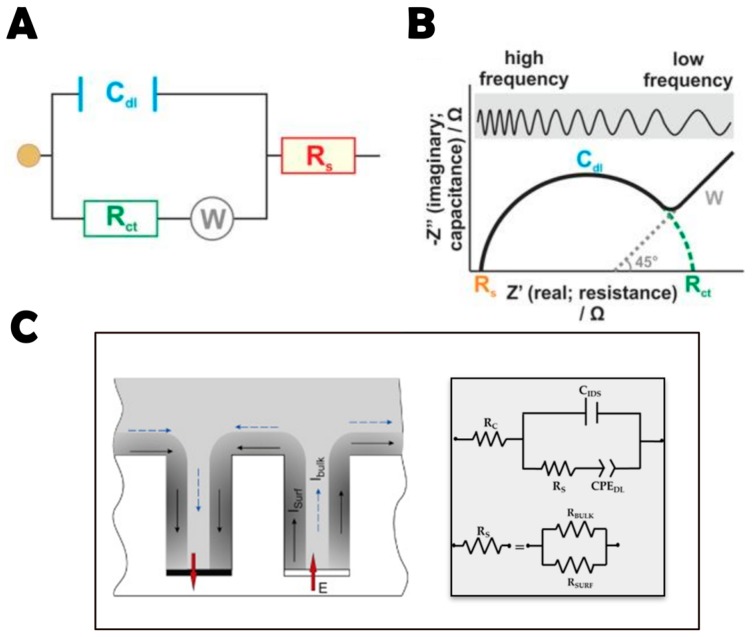
Data from EIS represented by (**A**) the Randles circuit; and (**B**) the Nyquist plot showing the features of the Randles circuit. It illustrates the components of the system: double-layer capacitance (C_dl_), charge transfer resistance (R_ct_), solution resistance (R_s_), and Warburg impedance (W). It should be noted that the Nyquist plot R_ct_ represents the semicircle diameter, so the right end of the semicircle indicates R_ct_+R_s_ and not R_ct_. Reprinted from [4], with permission from American Society for Microbiology. Copyright © 2014. (**C**) The electrical equivalent circuit used for impedance spectra fitting in IDEA surfaces in low conductivity KCl solutions in the absence of faradaic processes. Reprinted from [104], with permission from Elsevier. © 2016 Elsevier B.V.

EIS experimental data can be modeled with an electrical equivalent circuit (EC), which consists of resistances and capacitances combined in parallel or serially, as required, that represent different physicochemical properties [118]. The most used is the so-called Randles circuit (Figure 4A). The values of the electrical components are extracted from the equivalent electrical model using least-squares minimization fitting of the EIS spectrum [119]. Checking the variation of impedance components related to the system properties (e.g., solution composition), it is possible to compare impedance changes to individual EC components to verify the accurate selection of the EC [120].

To graphically analyze the data, the most common formats are the Bode and Nyquist plots. In these graphs the logarithm of the absolute impedance (log |Z|) and the phase shift (φ) are represented as a function of the logarithm of the excitation frequency f. In particular, in Nyquist plots, data are represented as the real component of impedance (Zre) on the *x* axis, and the imaginary component (Zim) on the *y* axis (Figure 4B) [4].

Different behaviors are obtained depending on the frequency range. For low frequency values, the dominant effect is ion diffusion (Warburg impedance) and the plot is represented by a straight line with a slope of 45°. On the other hand, at high frequencies the plot is described by a semi-circle with a diameter that is given by the charge transfer resistance Rct—owing to a bigger value of diffusion time constant compared to the signal period. Rct is the parameter most used to estimate bacterial concentration: when bacterial cells bind to the bioreceptors at the surface of the working electrode, the redox reaction is hindered and Rct increases [105]. Sometimes, however, the double layer capacitance Cdl is used instead [115]. 

In terms of bacterial detection, capacitive sensors such as interdigitated electrodes (IDEAs) have gained special interest in the field in recent years [121,122]. 

These transducers combine a series of advantages in comparison with other impedance-based sensors, including rapid kinetics of detection, improved signal-to-noise ratio, quick establishment of a steady-state response, low cost, and ease of miniaturization [118,119,120].

In a three-dimensional IDEA sensor, the general equivalent circuit that emulates the sensor impedance should be represented by the following components (Figure 4C): R_C_ is the contact resistance introduced by wires and collector bars of the thin film electrodes; C_IDS_ is the geometrical (stray) capacitance between two electrodes; R_S_ is the resistance between two electrodes of the array; and CPE_DL_ is a constant phase element representing the capacitance of the electrical double layer at the electrode-water solution interface. It has been previously reported that in low conducting solutions, surface conductivity plays an important role in this kind of sensors [114]. Therefore, R_S_ is a parallel combination of bulk solution resistance (R_BULK_) and the surface resistance (R_SURF_) (Figure 4C). It is important to note that under experimental conditions it is often not possible to distinguish these two elements in the impedance spectra. However, it is possible to fix the bulk solution conductivity and attribute the changes in R_S_ to surface resistance [25,114]. 

In summary, EIS represents an excellent technique for an accurate and sensitive biosensing of bacteria. On top of the advantages previously described, EIS does not require the use of labeling agents, and as such can be used to monitor bacterial binding on real-time. As a result of that, at present EIS is considered one of the most promising electrochemical techniques, and its number of applications in biosensing are rapidly increasing [123]. The final part of this review will focus on the most relevant examples of this method to detect bacteria. 

### 3.3. Examples of AMP-Based Biosensors for Bacterial Detection 

In this section, relevant examples of bacterial detection are presented (Table 2). We will report a few early studies using fluorescence spectroscopy (optical transducers) but the majority of works will focus on impedimetric biosensors (EIS technique). 

To the best of our knowledge, one of the first studies using an AMP as biorecognition element was reported by Kulagina et al. in 2005 [94]. In this study, the authors described a fluorescence-based biosensor functionalized with the AMP magainin I targeting *Escherichia coli* (*E. coli*) O157:H7 and *Salmonella typhimurium* (*S. typhimurium*), which are both considered among the most dangerous food-borne pathogens worldwide. The direct binding of magainin I on the sensor surface resulted in LoD of 1.6 × 10^5^ and 6.5 × 10^4^ cells/mL for *E. coli* and *S. typhimurium*, respectively (Table 2). In contrast, the immobilization of the peptide using biotin rendered sensors with lower affinity, thus indicating the importance of an appropriate presentation of the AMP for the sensing activity. 

In a subsequent study, this research group explored the use of a series of AMPs towards the same two bacterial strains. In detail, in addition to magainin I, cecropin A, parasin, polymyxin B and polymyxin E were immobilized on silanized glass slides at different peptide densities (Table 2) [91]. Interestingly, the AMPs displayed different degrees of affinity for the two bacteria (Table 3). It was also notable that the majority of the peptides did not interact with non-pathogenic *E. coli*. These results highlighted the fact that AMPs can be used to discriminate between different bacterial species, and even between strains of the same species. 

Another example following this approach evaluated a variety of AMPs (cecropin (A, B, and P), parasin, magainin I, polymyxin (B and E), melittin, and bactenecin) for the biodetection of Cy3-labeled Venezuelan equine encephalitis virus (VEE), vaccinia virus, *Coxiella burnetti* (*C. burnetti*) and *Brucella melitensis* (*B. melitensis*) (Table 2) [92]. The majority of the immobilized AMPs detected VEE, vaccinia virus and *C. burnetti* in a concentration-dependent manner, whereas *B. melitensis* preferably bound to polymyxin B, polymyxin E, and bactenecin. No binding of any pathogen was observed on immobilized magainin I. This work thus further strengthens the notion that AMPs may exert selectivity within distinct pathogens. 

Although these studies overall showed good affinity towards different bacterial strains, the LoD obtained were in a range comparable with antibody-based assays. Moreover, these studies relied on fluorescence microscopy, which implies the labeling of the bioreceptors/analytes prior to analysis. As previously introduced, impedimetric biosensors represent a powerful label-free alternative to reduce detection times and improve LoD in bacterial biosensing. As bacteria are generally electrically charged, when they are immobilized on the electrode surface they produce variations in the electrical impedance of the system. Bacterial attachment also implies a variation in interfacial impedance due to changes in surface conductivity produced by their electrical charge or the surface layer capacitance. Direct label-free impedance methods, which do not depend on bacterial growth or the production of metabolites, provide very fast detection times and will be presented in this section [120,124].

**Figure 5:**
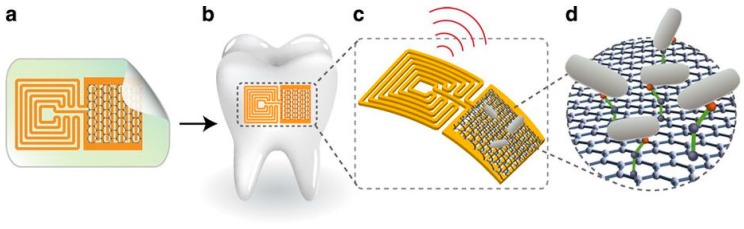
Graphene-based sensor for the remote (wireless) detection of bacteria. (**a**) Schematic representation of the nanosensor, containing a graphene/silk-based biosensor coupled to a resonant wireless coil; (**b**) Transfer of the nanosensor onto a tooth surface; (**c**) Illustration of the wireless signal upon bacterial detection; (**d**) Magnification of the bacterial interaction with the AMP-coated graphene platform. Reprinted by permission from Springer Nature-Nature Communications, from [98]. © 2012 Macmillan Publishers Limited.

Seminal experiments with electrochemical non-Faradic impedance technique with an AMP immobilized on IDEAs were presented by Mannoor et al. in 2010 [97]. In this study, magainin I was immobilized on gold microelectrodes via its C-terminal cysteine residue, and its capacity to bind bacterial cells was evaluated by EIS. To this end, the biosensor was exposed to concentrations of bacteria ranging from 10^3^ to 10^7^ CFU/mL. The variation in impedance was observed to be directly proportional to the number of bacterial cells bound to the immobilized AMPs. Of note, the LoD of the biosensor for *E. coli* O157:H7 was 10^3^ CFU/mL (approx. 1 bacterium/µL). Other bacterial species were tested to investigate the selectivity of the AMP-functionalized microelectrodes: Gram-negative pathogenic (O157:H7) and non-pathogenic (ATCC 35218) *E. coli*, Gram-negative *S. typhimurium* and Gram-positive *Listeria monocytogenes* (*L. monocytogenes*). Interestingly, the response of the biosensor was clearly preferential towards pathogenic Gram-negative species of *E. coli* and *S. typhimurium*, demonstrating the specificity of magainin I for these two pathogenic bacteria, in agreement with previous reports [92,94]. The same research group described a few years later a wireless graphene-based nanosensor integrated on a tooth for remote monitoring of respiration and bacteria detection in saliva (Figure 5) [98]. The biosensing unit was a peptide construct based on a graphene-binding peptide (GBP) and the AMP odorranin-HP (OHP). 

Ferrocene (Fc) and its derivatives are often used in electrochemical systems owing to their beneficial electrochemical properties [125]. In this regard, Li et al. described a novel biosensor composed of the conjugate Fc-magainin I on a gold electrode for the detection of pathogenic *E. coli* O157:H7 [101]. Non-pathogenic of *E. coli* K12, *Staphylococcus epidermidis* (*S. epidermidis*) and *Bacillus subtilis* (*B. subtilis*) were also included in this study to evaluate the selectivity of the biosensor. As observed in previous studies [92,94,97], magainin I showed selectivity for pathogenic *E. coli*. Accordingly, the sensor displayed the following trend of selectivity: pathogenic *E. coli* O157: H7 (LoD of 10^3^ CFU/mL) > non-pathogenic *E. coli* > the two Gram-positive species. A similar LoD (1.5 × 10^3^ CFU/mL) for *E. coli* O157: H7 was obtained using a synthetic AMP (GIGKFLHSAGKGKAFVGEIMKSC) coated on a gold electrode via its C-terminal cysteine residue (Table 1) [95]. Interestingly, the sensor could be regenerated and used up to 20 times, maintaining almost 80% of the signal response obtained on the first measurement and thus demonstrating the good stability of the biosensing AMP.

As we commented before, the sensitivity and specificity of AMPs may decrease if the detection is done in real samples, such as blood or milk. To overcome false positive signals from the non-specific binding of proteins and others biomolecules present in food samples, Etayash et al. reported a new impedance array analyzer that works at very low frequency to detect Gram-positive bacteria [100]. The AMP used was leucocin A, a naturally occurring AMP from class IIa bacteriocins, which possesses high antibacterial potency a Gram-positive species such as *L. monocytogenes*. In detail, the AMP was immobilized on interdigitated gold microelectrodes via its C-terminal carboxylic acid and was capable of selectively detecting *L. monocytogenes* in contaminated milk samples with a LoD of 10^3^ CFU/mL. In another recent study, a microfluidic chip based on an electrical impedance microsensor array functionalized with two species-specific synthetic AMPs (C16G2cys or G10KHc) was described [99]. Peptide immobilization on the surfaces was made via cysteine–gold interactions, and the resulting biosensors efficiently detected *Streptococcus mutans* and *Pseudomonas aeruginosa* (*P. aeruginosa*) at minimum concentrations of 10^5^ CFU/mL in only 25 min. 

To further improve the sensitivity (i.e., LoD) of impedimetric sensors, several recent approaches have been described. For example, Liu et al. designed a multidomain AMP with the sequence WK_3_(QL)_6_K_2_G_3_C for highly sensitive bacterial detection [103]. The antimicrobial activity of this peptide was dependent on its conformation, which was a mixed of random coil (WK_3_) and beta-sheet ((QL)_6_K_2_) secondary structures. The peptide was bound to the gold electrodes via its C-terminal moiety (G_3_C). This peptide allowed the detection of *E. coli*, *P. aeruginosa*, *Staphylococcus aureus* (*S. aureus*) and *S. epidermidis* with a LoD of only 10^2^ CFU/mL. 

**Figure 6:**
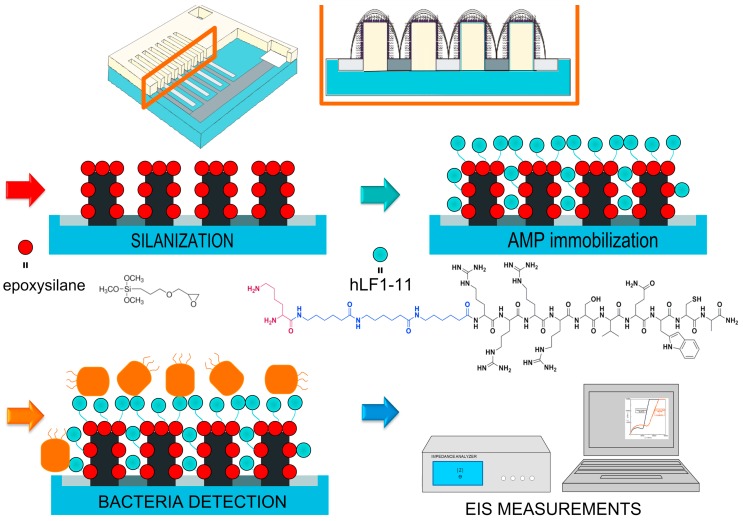
Schematic representation of the biosensor design and its functionalization with the hLF1-11 peptide. 3D-IDEA electrodes were silanized in vapor phase with an epoxysilane. The hLf1-11 peptide contains 3 parts: (i) an antibacterial domain (black), (ii) a spacer unit (blue), and (iii) a lysine residue that acts as anchoring moiety (violet). The binding of *S. sanguinis* to the AMP-coated surface is measured by EIS technique. Reprinted from [104], with permission from Elsevier. © 2016 Elsevier B.V.

Another strategy is to use three-dimensional IDEA (3D-IDEA) devices [126], which have insulating barriers separating the electrode digits and permit to considerably enhance the sensitivity of the transducer compared to conventional (flat) IDEA sensors. The 3D geometry translates into a higher capacity to monitor changes on the surface charge when a target molecule binds to the sensor. In this regard, we recently reported the combination of miniaturized and integrated 3D-IDEA and the AMP hLf1-11 for the impedimetric detection of periodontopathogenic bacteria (Figure 6) [104]. The peptide hLf1-11 was chosen for its well-known activity against *Streptococcus sanguinis* (*S. sanguinis*) a primary colonizer in oral biofilms, as reported by us in several studies [19,127,128,129] and immobilized on the biosensor using vapor-phase silanization. The developed biosensors very efficiently detected *S. sanguinis* in both KCl samples (LoD: 3.5 × 10^1^ CFU/mL) and artificial saliva (LoD: 8.6 × 10^2^ CFU/mL) at very short detection times (30 min). Of note, such low LoD are uncommon for these types of sensors, especially in complex samples such as saliva (Table 2).

Finally, a third approach to improve the LoD of AMP-based impedimetric sensors is to use nanomaterials as signal amplifiers (see Section 2.3 for details) [123,130,131]. For instance, Andrade et al. recently developed a detection system combining CNT and the AMP clavanin A [102]. These nanostructured sensors were able to detect bacteria in a wide range of concentrations, 10^2^–10^6^ CFU/mL. Specifically, the biosensors showed LoD of 10^2^ CFU/mL for *E. coli* and *Klebsiella pneumoniae* (*K. pneumonia*) and of 10^3^ CFU/mL for *Enterococcus faecalis* (*E. faecalis*) and *B. subtilis*. Moreover, this system was able to differentiate between Gram-positive and Gram-negative, and between pathogenic and non-pathogenic bacteria. In subsequent studies, this research group used the same AMP, clavanin A, conjugated to gold nanoparticles [105]. Noteworthy, this method allowed a linear range of detection from 10^1^ to 10^4^ CFU/mL and a LoD of only 10 CFU/mL for *S. aureus*, *E. faecalis*, *P. aeruginosa*, *S. typhimurium* and *E. coli.* Such LoD is one of the lowest values described so far for AMP-based impedimetric sensors in the literature (Table 2).

## 4. Conclusions

The field of biosensing is advancing rapidly and it is expected to provide technological solutions to new challenges of our society. One of such challenges is the growing emergence of bacterial resistance and the difficulty in treating pathogenic biofilms. In this regard, biosensors allow a rapid, cost-effective and specific detection of bacterial cells, and thus will facilitate the identification and treatment of infections at very early stages.

This review has particularly focused on the use of impedimetric biosensors containing AMPs as biorecognition elements. AMPs have shown unique properties in comparison with other “classical” bioreceptors such as enzymes or antibodies, including high sensitivity, stability and ease of manufacturing. Moreover, we have shown that AMPs are also capable of achieving good levels of specificity, discriminating between pathogenic and non-pathogenic bacteria or between Gram-positive and Gram-negative species. In addition, we have shown that the sensitivity of AMP-based biosensors can be finely tuned and improved by different strategies like the use of signal amplifiers, 3D-electrodes or rationally engineering of the peptidic structure. Such approaches resulted in LoD as low as 10 CFU/mL, even in complex samples like saliva in very short detection times. 

We have shown that AMP-impedimetric sensors combine excellent properties to produce robust, label-free, rapid, sensitive, specific and cost-effective platforms for the early detection of bacterial infections. However, the number of studies in this area is still limited. It is foreseen that more research will shed light on the opportunities of these systems for technological transfer and its actual use in clinical settings. 

## Figures and Tables

**Table 1:** Examples of different combinations between biorecognition elements and transducers to obtain biosensors for bacterial detection.

		Transducer		
		Optical	Mechanical	Electrochemical
	Adhesin (Ad) or None	---	Ad + Nanowires [18]	---
**Bioreceptor**	**Enzyme (Ez)**	(i) Ez NP + colorimetric [43] (ii) Bacteria-specific Ez + colorimetric [44]	---	Ez NP + DPV [45]
	**Antibody (Ab)**	(i) Ab + MNP + SPR [46] (ii) Ab + SPR [47]	(i) Ab + QCM [48] (ii) Ab + nanobeads + QCM [49] (iii) Au NP + Ab + QCM [50,51] (iv) Ab + cantilever [52] (v) Ab + PEMC [53]	(i) Ab + amperometric [54,55] (ii) Ab+ impedimetric [14,56,57,58,59,60] (iii) Ab MNP + DPV [61] (iv) Ab Mbeads + amperometric [54,55,62]
	**DNA/RNA/** **Aptamer (Ap)/Aptazyme (Apz)**	(i) Ap + colorimetric [63,64] (ii) Ap + LSPR [65] (iii) Apz + Mbeads + colorimetric [66]	(i) thssDNA + QCM [67] (ii) ssDNA + cantilever [68]	(i) Ap + potentiometric [69,70] (ii) Ap + impedimetric [71]
	**Lectin (L)**	L + SPR [72]	L + piezoelectric [73,74]	L + impedimetric [75,76,77]
	**Phage (Ph)**	(i) Ph + SPR [78,79] (ii) Ph + MES + colorimetric [80] (iii) Ph + Mbeads + colorimetric [81]	(i) Ph + QCM [82] (ii) Ph + MES [83,84,85,86]	(i) Ph + amperometric [87] (ii) Ph + impedimetric [57,88,89,90]
	**AMP**	AMP + fluorescence spectroscopy [91,92,93,94]	AMP + QCM [95,96]	AMP + impedimetric [95,97,98,99,100,101,102,103,104,105]

MES: magnetoelastic sensor; NP: nanoparticle; MNP: magnetic NP; SPR: surface plasmon resonance; LSPR: Localized SPR; QCM: quartz crystal micro-balance; AMP: antimicrobial peptide; PEMC: piezoelectric-excited millimeter-size cantilever; DPV: differential pulse voltammetry; Mbeads: magnetic beads; thssDNA: thiolated single-stranded DNA; ssDNA: single-stranded DNA.

**Table 2:** AMP-based sensors for bacterial detection.

AMP Sequence	Bacteria Detected	LoD (CFU/mL)	Application	Type of Transd.	Sensor Structure	Detection Time	Ref.
Magainin I	*E. coli* O157:H7; *S. typhimurium*	1.6 × 10^5^; 6.5 × 10^4^ cells/mL	---	FS	Silanized glass slide on array-based biosensor	70 min	[94]
Cecropin A, parasin, magainin I, polymyxin B and E	*E. coli* O157:H7; *S. typhimurium*	See Table 3	Detection of foodborne contaminants	FS	Glass slides-PDMS on a mixed “sandwich” assay (multi-AMP array)	---	[91]
Cecropin (A, B, and P), parasin, magainin I, polymyxin (B and E), melittin, bactenecin	*C. burnetti*; *B. melitensis*; VEE; vaccinia virus	5 × 10^5^ cells/mL; 5 × 10^4^; <5 × 10^5^ cells/mL; <5 × 10^5^	Detection of inactivated targets of biodefense interest	FS	Glass slides-PDMS on array-based biosensor	---	[92]
Magainin I	*E. coli* O157:H7; *S. typhimurium*	10^3^	Detection of an infectious outbreak from a broad spectrum of pathogenic species	EIS	Gold surface-cysteine on IDEA	---	[97]
GBP + OHP	*E. coli*; *H. pylori*; *S. aureus*	10^3^ in wireless operation mode	Duodenal ulcers and stomach cancers	EIS	IDEA with graphene resistive sensors in a silk support	---	[98]
Magainin I	*E. coli* O157:H7; *E. coli* K12; *B. subtilis*; *S. epidermis*	10^3^	Life-threatening gastrointestinal infections	EIS	Ferrocene-Magainin conjugate on a gold electrode	---	[101]
Leucocin A	*L. monocytogenes*; *S. aureus*	10^3^	---	EIS	Gold surface- cysteamine on IDEA	20 min	[100]
G10KHc, C16G2cys	*P. aeruginosa*; *S. mutans*	10^5^	Infectious diseases	EIS	Gold surface-cysteine on microfluidic chip	25 min	[99]
Clavanin A	*K. pneumoniae*; *E. faecalis*; *E. coli*; *B. subtilis*	10^2^; 10^2^; <10^3^; <10^3^	Detect pathogens with high resistance to conventional antibiotics	EIS	Nanostructured sensor based on carbon nanotubes on gold electrode	---	[102]
GIGKFLHSAGKGKAFVGEIMKS	*E. coli* O157:H7	1.5 × 10^3^	Bacterial infections	EIS	Mixed self-assembled monolayer on a three electrode system	30 min	[95]
WK3(QL)6K2G3C	*E. coli*; *P. aeruginosa;* *S. aureus*; *S. epidermidis*	10^2^	Bacterial infections	EIS	Gold disk electrode	---	[103]
hLF1-11	*S. sanguinis*	KCl: 3.5 × 10^1^; AS: 10^2^	Bacterial infections	EIS	3D-IDEA based on silicon dioxide insulating substrate	30 min	[104]
Clavanin A	*E. coli*; *S. typhimurium*; *E. faecalis*; *S. aureus*	~ 10	Dental infections	EIS	AuNPsCys	70 min	[105]

AS: artificial saliva; EIS: electrochemical impedance spectroscopy; FS: fluorescence spectroscopy; AuNPsCys: cysteine-modified gold nanoparticles; PDMS: polydimethylsiloxane; VEE: Venezuelan equine encephalitis virus; GBP: graphene-binding peptide; OHP: odorranin-HP.

**Table 3:** Limits of detection (LoD) (CFU/mL) of different types of AMPs for the detection of *E. coli* and *S. typhimurium.*

AMP	*E. coli*	*S. typhimurium*
Polymyxin B	1 × 10^5^	5 × 10^5^
Polymyxin E	5 × 10^5^	5 × 10^6^
Magainin	5 × 10^4^	1 × 10^5^
Cecropin A	1 × 10^5^	5 × 10^5^
Parasin	5 × 10^5^	1 × 10^6^

Data from [91].
